# The FOCUS, AFFINITY and EFFECTS trials studying the effect(s) of fluoxetine in patients with a recent stroke: a study protocol for three multicentre randomised controlled trials

**DOI:** 10.1186/s13063-015-0864-1

**Published:** 2015-08-20

**Authors:** Gillian Mead, Maree L. Hackett, Erik Lundström, Veronica Murray, Graeme J. Hankey, Martin Dennis

**Affiliations:** Centre for Clinical Brain Sciences, University of Edinburgh, Chancellors Building FU303h, 49 Little France Crescent, Edinburgh, EH16 4SB UK; The George Institute for Global Health, University of Sydney, Sydney, Australia; Department of Neurology, Karolinska University Hospital, Solna, Sweden; Karolinska Institutet, Stockholm, Solna Sweden; School of Medicine and Pharmacology, University of Western Australia, Perth, Western Australia Australia

**Keywords:** Ischaemic stroke, Haemorrhagic stroke, Antidepressants, SSRI, Fluoxetine, Recovery, Depression

## Abstract

**Background:**

Several small trials have suggested that fluoxetine improves neurological recovery from stroke. FOCUS, AFFINITY and EFFECTS are a family of investigator-led, multicentre, parallel group, randomised, placebo-controlled trials that aim to determine whether routine administration of fluoxetine (20 mg daily) for 6 months after acute stroke improves patients’ functional outcome.

**Methods/Design:**

The three trial investigator teams have collaboratively developed a core protocol. Minor variations have been tailored to the national setting in the UK (FOCUS), Australia and New Zealand (AFFINITY) and Sweden (EFFECTS). Each trial is run and funded independently and will report its own results. A prospectively planned individual patient data meta-analysis of all three trials will subsequently provide the most precise estimate of the overall effect of fluoxetine after stroke and establish whether any effects differ between trials and subgroups of patients.

The trials include patients ≥18 years old with a clinical diagnosis of stroke, persisting focal neurological deficits at randomisation between 2 and 15 days after stroke onset. Patients are randomised centrally via web-based randomisation systems using a common minimisation algorithm. Patients are allocated fluoxetine 20 mg once daily or matching placebo capsules for 6 months. Our primary outcome measure is the modified Rankin scale (mRS) at 6 months. Secondary outcomes include the Stroke Impact Scale, EuroQol (EQ5D-5 L), the vitality subscale of the Short-Form 36, diagnosis of depression, adherence to medication, adverse events and resource use. Outcomes are collected at 6 and 12 months. The methods of collecting these data are tailored to the national setting. If FOCUS, AFFINITY and EFFECTS combined enrol 6000 participants as planned, they would have 90 % power (alpha 5 %) to detect a common odds ratio of 1.16, equivalent to a 3.7 % absolute difference in percentage with mRS 0–2 (44.0 % to 47.7 %). This is based on an ordinal analysis of mRS adjusted for baseline variables included in the minimisation algorithm.

**Discussion:**

If fluoxetine is safe and effective in promoting functional recovery, it could be rapidly, widely and affordably implemented in routine clinical practice and reduce the burden of disability due to stroke.

**Trial registration:**

FOCUS: ISRCTN83290762 (23/05/2012), AFFINITY: ACTRN12611000774921 (22/07/2011). EFFECTS: ISRCTN13020412 (19/12/2014).

**Electronic supplementary material:**

The online version of this article (doi:10.1186/s13063-015-0864-1) contains supplementary material, which is available to authorized users.

## Background

### The burden of stroke

Each year, stroke affects 16 million people for the first time and causes about 5.7 million deaths worldwide [1]. About 50 % of survivors will have long-term residual disability. This places a huge burden on health and social services and informal carers. Although there is more that can be done to implement effective treatments such as thrombolysis and more rapid access to stroke units, there is still an urgent need to identify new treatments that might reduce neurological impairments, disability and dependency. One promising intervention that needs to be tested is a widely used antidepressant drug, fluoxetine, a selective serotonin reuptake inhibitor (SSRI).

**Fig. 1:**
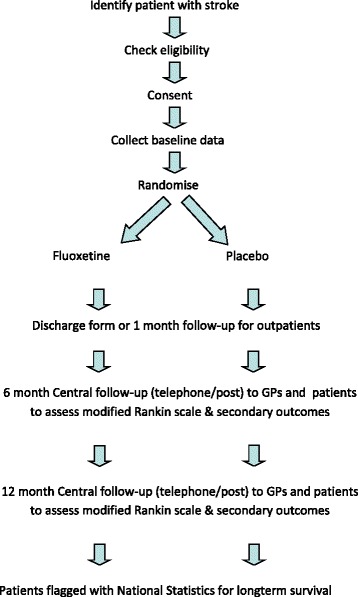
Patient flow in the FOOD, AFFINITY and EFFECTS trials

### SSRIs

SSRIs have been used in clinical practice since 1988 to treat mood disorders, particularly depression. They are sometimes used to manage emotionalism after stroke. Furthermore, animal studies have shown that fluoxetine may attenuate post-ischaemic brain injury by facilitating expression of neuro-protective and regenerative proteins, suppressing post-stroke hyperexcitability in unaffected brain and reducing inflammation [2, 3]. SSRIs may also stimulate neuronal generation [4, 5], secretion of growth factors that augment neuroplasticity [6, 7], synaptic plasticity [8], expression of brain phosphorylated cyclic adenosine monophosphate response element binding protein [9] and attenuate hypothalamic pituitary axis overactivity [10], thus reducing cortisol, which is associated with poorer outcomes post-stroke [11].

A systematic review of SSRIs in animal models of stroke identified 21 experiments reporting the efficacy of fluoxetine in 252 animals; neurobehavioural scores improved by 41 % (95 % CI 27–54 %) but there was insufficient evidence to determine the likely underlying mechanisms [12].

In healthy humans, functional magnetic resonance imaging studies have demonstrated that fluoxetine can modulate cerebral motor activity [13]. In strokes resulting in motor deficits, fluoxetine can cause hyperactivation in the ipsi-lesional primary motor cortex during a motor task [14] and a decrease of motor excitability over the unaffected hemisphere [15].

### Promising effect of fluoxetine on stroke recovery in the fluoxetine for motor recovery after acute ischaemic stroke (FLAME) trial [16]

The FLAME trial results, presented in October 2010, ignited worldwide interest in the role of fluoxetine for motor recovery [16]. In this double-blind, placebo-controlled, multicentre trial 118 patients with ischaemic stroke and unilateral motor weakness were randomised to receive fluoxetine 20 mg daily or placebo for 3 months. At day 90, the improvement in the Fugl Meyer motor score from baseline was significantly greater in the fluoxetine group. Also, the frequency of independent patients [with a modified Rankin scale (mRS) of 0–2] was significantly higher in the fluoxetine group (26 % vs. 9 %, *p* = 0.015) although there were not significant differences at other mRS cut-offs.

### Cochrane systematic review of SSRIs for stroke recovery [17]

In a Cochrane systematic review of SSRIs for stroke recovery we identified 52 randomised controlled trials of SSRI versus control (4059 patients), given within the first year after ischaemic or haemorrhagic stroke, for any indication [17]. Only two trials (*n* = 223), including FLAME, reported the mRS as an outcome measure; the proportion of patients with mRS 0–2 at the end of treatment was 62.5 % in the SSRI group and 55 % in the control group (absolute difference 7.5 %, 95 % CI: −5.3 to 20.4). Among 22 trials (1310 patients) that reported disability as an outcome measure, meta-analysis demonstrated a large effect size of SSRI (SMD 0.92, 95 % confidence intervals 0.62 to 1.23). The effect size was larger for patients with depression at recruitment [standardised mean difference (SMD) 1.11, 95 % CI 0.71, 1.51] than those without (SMD 0.55, 95 % CI 0.27 to 0.84) (*p* = 0.03 between groups). The effect size was smaller in trials at low risk of bias. SSRIs improved several secondary outcomes (e.g. neurological impairment, depression and anxiety) at the end of treatment, but there was a non-significant excess of seizures, gastrointestinal adverse effects and bleeding. However, the meta-analysis identified multiple biases in trial design, substantial statistical heterogeneity among the trials, wide confidence intervals for effect estimates and limited data on adverse effects. Only seven trials followed up patients after treatment had ended; of these only two (*n* = 155) provided data on disability.

Fluoxetine was the most commonly used drug in the review. However, only 12 fluoxetine trials (*n* = 682) were placebo controlled. Of these, six measured the degree of functional recovery; fluoxetine was associated with less disability compared with placebo (SMD 0.35, 95 % CI 0.03 to 0.61) at the end of treatment. Although promising, the data are not sufficiently compelling to prove that fluoxetine improves functional recovery after stroke and that any possible benefits are not offset by serious adverse effects.

### Why choose fluoxetine to test in a large randomised trial?

We have chosen to evaluate fluoxetine because it is one of the most widely studied SSRIs. Its safety profile is very well established, and the drug is well tolerated, in long-term use, even in older people. There are more trials in stroke, and fewer concerns about safety in the elderly and in patients with cardiovascular disease, than for alternatives, such as citalopram [17]. A number of manufacturers produce the drug and the price is low. Lastly, of all the SSRIs, it has the longest half life, so that gradual reduction in dose is not required when withdrawing the drug (which is inevitable in a trial) to reduce the possibility of an SSRI withdrawal syndrome [18].

### What are the potential risks of fluoxetine in stroke?

There are potential risks associated with giving fluoxetine to a wide range of stroke patients. Our systematic review indicated that SSRIs, compared with placebo or usual care, were associated with a non-significant excess of seizures [relative risk (RR) 2.7; 95 % CI 0.6-11.6] (7 trials, 444 participants), gastrointestinal adverse effects (RR 1.9; 0.9-3.8) (14 trials, 902 participants) and bleeding (RR 1.6; 0.2-13) (2 trials, 249 participants) [17]. Cohort studies, whilst prone to confounding and indication bias, have also reported that SSRI use is associated with increased risk of seizures, bleeding and hyponatraemia, particularly during the first 4 weeks of treatment [19–23]. Its interaction with antiplatelet and anticoagulant medication might increase bleeding risk. Hence, co-prescription of fluoxetine with antiplatelet or anticoagulant medications that are commonly used by stroke patients might increase bleeding risk in this population of patients. Like other antidepressants, fluoxetine may lower seizure threshold and therefore could increase the frequency of post-stroke seizures. Although evidence on this is conflicting, we are excluding patients with a history of epileptic seizures [24]. An adverse effect on glycaemic control in diabetics has been recorded. Hyponatraemia is a recognised adverse effect and may prove to be more common amongst stroke patients who may be taking concomitant angiotensin-converting enzyme inhibitors, diuretics and proton pump inhibitors. However, reassuringly, fluoxetine has been very commonly prescribed for several years to patients with stroke to treat depression and emotionalism without major problems emerging. Subject to assessment by the responsible clinician, some stroke patients with severe renal or hepatic failure may not be able to participate in the trials.

### The need for large randomised trials of fluoxetine in stroke

A cardinal principle of all research should be “trust but verify”. Given the encouraging data from the FLAME trial and other smaller studies, there is an urgent need to carry out randomised trials that have adequate power to reliably detect clinically important benefits and hazards. Fluoxetine is inexpensive (only about £2.50 per month in the UK), simple-to-administer and generally well tolerated. If it proves to be safe in stroke patients and has an effect on functional recovery after stroke that is even a fraction of that seen in the FLAME trial it would be a very worthwhile treatment for patients, their carers, and health and social services.

### The need to identify the patients who might particularly benefit from treatment

Whilst fluoxetine may improve outcome for the whole range of stroke patients, it is also plausible given its diverse pharmacological effects that the balance of risk and benefit may vary in patients with different types of stroke. For instance, pre-clinical work has suggested that motor recovery may be specifically enhanced (see above). Also, fluoxetine influences bleeding risk, particularly in those taking antithrombotic medication, so there could be differences in effectiveness between patients with ischaemic (who are taking antithrombotics) and those with haemorrhagic stroke. Patients with severe stroke associated with cognitive and communication problems may be at greater risk of adverse effects because patients are unable to report early problems but they might also have more to gain from a treatment that enhances recovery. Also, those with severe stroke are normally at greater risk of post-stroke depression (which is associated with stroke severity) but—as a consequence of their deficits—are at greater risk that their post-stroke depression is not recognised and so goes untreated [25].

#### Study objectives

We have collaboratively designed and implemented a family of three large, investigator-led, government and charity-funded, multicentre, placebo-controlled randomised trials that together aim to robustly address several research questions.

Our aims are to determine whether the routine administration of fluoxetine 20 mg od started between 2 and 15 days post stroke, and continued for 6 months, improves recovery and whether any benefits persist after the treatment has stopped until 12 months after the stroke.

### Primary research question

Does the routine administration of fluoxetine (20 mg od) for 6 months after an acute stroke improve patients’ functional status at 6 months?

### Secondary research questions

1. If fluoxetine improves functional status at 6 months, does any improvement in functional status persist after treatment is stopped?
2. Does fluoxetine influence the secondary outcome measures (stroke impact, fatigue, mood and quality of life) at 6 months and 12 months?
3. Does fluoxetine increase the risk of serious adverse events?
4. If fluoxetine is effective, is it also cost-effective?
5. Is fluoxetine associated with longer-term survival? Functional outcome at 6 months post stroke is strongly associated with long-term survival and so we wish to determine whether any benefits on functional outcome would be translated into longer-term survival.
6. Does the presence or absence of any of the following factors materially alter the effect of fluoxetine on our primary outcome?
  - Stroke pathology (i.e. haemorrhagic stroke vs. ischaemic stroke)
  - Age (age ≤70, >70 years)
  - Stroke severity (predicted probability of a good outcome vs. poor outcome)
  - Depression at baseline
  - Inability to assess mood because of communication or cognitive problems (based on need for proxy consent)
7. In patients with motor deficits at randomisation does fluoxetine improve motor function?
8. In patients with aphasia at randomisation does fluoxetine improve communication?
9. Is there a relationship between functional status at 6 months and mood and is this relationship affected by fluoxetine?

## Methods

### Design

The FOCUS, AFFINITY and EFFECTS trials are multicentre, parallel-group, double-blind, placebo-controlled trials with broad entry criteria and follow-up to ascertain the primary and secondary outcomes at about 6 and 12 months (Fig. 1). This section describes the core protocol that the three trials share and the variations adopted to facilitate each trial in its national setting. The following description reflects the versions of the trial protocols in use on 1 March 2015.

### Start-up phases

Each trial has completed a start-up phase to establish whether the protocol is feasible in each setting and to establish the trial management teams, IT systems to manage web-based randomisation, drug allocation, stock control, follow-up, data collection and verification, and important aspects of feasibility including recruitment, medication adherence, questionnaire completion and follow-up rates.

### Main phase

The trials are powered to detect differences in a primary outcome based on an ordinal analysis of the seven-category modified Rankin scale (mRS 0, 1, 2, 3, 4, 5, 6) for the entire group [26]. Because it is not feasible to enrol sufficient patients in each trial to reliably detect small effect sizes that would still be of clinical significance we plan to perform an individual patient data meta-analysis including the data from FOCUS, AFFINITY and EFFECTS. This will allow us to provide the most precise estimates of any risks and benefits to detect a smaller overall effect size than those detectable by the individual trials and also to determine the effects in subgroups.

### Patient population

Patients will be identified by participating clinicians from in-patient stroke services and outpatient clinics in the UK (FOCUS), Australasia and Asia (AFFINITY) and Sweden (EFFECTS).

Our inclusion criteria are:

- Males and females aged ≥18 years
- A clinical stroke with brain imaging that is compatible with intracerebral haemorrhage or ischaemic stroke (including those with normal CT scans)
- Randomisation can be performed between 2 and 15 days after stroke onset
- Persisting focal neurological deficit is present at the time of randomisation severe enough to warrant treatment from the patient’s or carer’s perspective.

Our exclusion criteria are:

- Subarachnoid haemorrhage (except where secondary to a primary intracerebral haemorrhage or enrolling in AFFINITY)
- Unlikely to be available for follow-up for the next 12 months, e.g. no fixed home address
- Unable to speak English (FOCUS) or Swedish (EFFECTS) AND no close family member available to help with follow-up forms
- Other life-threatening illness (e.g. advanced cancer) that will make 12-month survival unlikely
- History of epileptic seizures
- History of allergy to fluoxetine
- Contraindications to fluoxetine including:
- Hepatic impairment (alanine aminotransferase >3 upper normal limit)
- Renal impairment (creatinine levels >180 micromol/l and in AFFINITY also eGFR <30 ml/min/1.73 m^2^)
- Hyponatraemia (sodium < 125 mmol/l) in AFFINITY
- Pregnant or breast-feeding women of child-bearing age not taking contraception. Minimum contraception is an oral contraceptive
- Previous drug overdose or attempted suicide
- Already enrolled into a CTIMP
- Current or recent (within the last month) depression requiring treatment with an SSRI antidepressant. AFFINITY also excludes patients requiring treatment or currently treated with any antidepressant
- Current use of medications that have a serious interaction with fluoxetine
- Use of a monoamine oxidase inhibitor (MAOI) during the last 5 weeks (e.g. phenelzine, isocarboxacid, tranylcypromine, moclobemide selegiline and rasagiline)
- Pimozide
- AFFINITY also excludes those taking tramadol unless the person is willing to stop
- AFFINITY specifically excludes patients with a diagnosis of bipolar disease and patients receiving treatment with an antipsychotic medication or tamoxifen
- EFFECTS excludes patients who are unable to consent for themselves, FOCUS and AFFINITY allow consent by a proxy, and AFFINITY allows waiver of consent in specific circumstances.

### Co-enrolment

Inclusion in another research study does not automatically exclude a patient from participating in these trials. As long as inclusion in the other study would not confound the results of the trials or make attribution of adverse reactions difficult, co-enrolment is permissible. However, if a participant has already been enrolled into another CTIMP, they cannot be enrolled into the trials. If a patient is enrolled into one of the trials, they may not subsequently be enrolled into another CTIMP. Also, local researchers must avoid overburdening patients.

### Randomisation

Having obtained consent, the randomising person collects the baseline data (see Table 1) on a randomisation form and enters these data into a trial-specific computerised central randomisation service by means of a secure 24/7 Web interface or a telephone call to the trial office during office hours. After the computer programme has checked these baseline data for completeness and consistency it allocates that patient a unique study identification number and a treatment pack number that corresponds to either fluoxetine or placebo. The trial-specific system applies a common minimisation programme to achieve a balance of four factors:

- Delay since stroke onset (2–8 vs. 9–15 days)
- Predicted 6-month outcome (based on the six simple variable model [27])
- Presence of a motor deficit [based on the National Institute of Health Stroke Scale (NIHSS)] [28]
- Presence of aphasia (based on NIHSS)

**Table 1:** Baseline data collected prior to randomisation in the three trials

Data item	FOCUS	AFFINITY	EFFECTS
Eligibility confirmed	+	+	+
Consent confirmed	+	+	+ Patient only
Participant’s names	+	+	+
Date of birth	+	+	+
Gender	+	+	+
Ethnicity	+	+	+
Living arrangements	+	+	+
Employment	+	+	+
Co-morbidities (existing)			
• Previous ischaemic stroke/TIA	+	+	+
• Previous intracranial bleeding	+	+	+
• Coronary heart disease	+	+	+
• Current or past depression	+	+	+
• Diabetes	+	+	+
• Gastrointestinal bleeding	+	+	+
• Hyponatraemia	+	+	+
• Fractures	+	+	+
Current medication	+	+	+
NIHSS including subsections [28]	+	+	+
Prior independence	1 Question	mRS	1 Question
Ability to walk alone	+	+	+
Ability to lift both arms	+	+	+
Post-stroke disability (smRSq) [31–33]		+	
Patient health questionnaire (PHQ) [43, 56]	2-Question version	9-Question version	2-Question version
Haemorrhage on brain imaging?	+	+	+
OCSP classification for ischaemic stroke [57]	+	+	+
Modified TOAST classification [58]	+	+	+
Renal and liver function test results		+	
Contact details to facilitate central follow-up	+		+
Unique identifier to facilitate central follow-up	+		

Following randomisation, the trial co-ordinating centre (FOCUS, EFFECTS) or randomising centre (AFFINITY) generates and sends a letter to inform the General Practitioner of the patient’s enrolment in the trial, including a copy of the consent form, follow-up schedule and advice about treatment of depression in patients participating in the trials.

### Treatment allocation

The minimisation algorithm randomly allocates the first patient to a treatment, but allocates each subsequent patient in a ratio of 1:1 to the treatment that leads to the least difference between the treatment groups with respect to the prognostic factors [29]. To ensure that we retain a random element to treatment allocation, patients are allocated to the group that minimises differences between groups with a probability of 0.8. The systems contain a list of treatment codes for each centre and that match the stock of IMP held at that centre. At the end of the session each patient is allocated a treatment code that corresponds to either an active (fluoxetine 20 mg once daily) or placebo treatment pack with a 6-month supply of capsules.

Patients are prescribed the study medication (20 mg capsule of fluoxetine or placebo capsule) to be taken daily at a time that is likely to maximise their adherence, i.e. linked to an activity of daily living. If the patient is unable to swallow capsules and has an enteral feeding tube in place then the capsules may be broken open and the contents put down the tube according to accepted methods [30].

### Blinding

The patient, their families, the healthcare team including the pharmacist and anyone involved in outpatient assessments are blinded to the treatment allocation. Emergency unblinding systems are available for each trial. The relevant chief investigators will decide on a case-by-case basis whether unblinding is required to ensure patient safety.

### Follow-up

Participants are followed up in all three trials at 6 and 12 months to collect the primary and secondary outcomes. However, the trials vary in the timing, frequency and method of monitoring the patients’ progress (Table 2).

**Table 2:** Study assessment schedules

Assessment	Baseline	At discharge	1 Week	4 Weeks	12 Weeks	26 Weeks	30 Weeks	52 Weeks
Consent and randomise	x							
Contact details	x	f,e		a				
Living circumstances	x	f		x	e	x		x
Training (physio, etc.)			e	e		e		
1^0^ Outcome-smRSq (mRS) [26, 31–33]	a			a		x		x
Depression diagnosis	x	f,e		a	x	x		x
PHQ2 [56]	f,e							
PHQ9 [43]	a			a		a		a
MHI 5 [37–39]						f,e		f,e
MADRS [40, 41]					e	e		e
Emotionalism						e		
DSM IV for depression [42]					e	e	e	e
SIS [34–36]						x		x
Fatigue subscale SF36 [44, 45]				x	e	x		x
Cognition (TICSm) [46]						a		a
Cognition (MoCA) [47]	e					e		
EQ5D-5 L [48]			e	e		x		x
EQD thermometer						a		a
SF12 [59]								a
Adverse events		f,e	e	a,e	e	x		x
Adherence to IMP		f	e	a,e	e	x		
All medications	x	f		a		x		a
Retrieve residual capsules (pill count)					e	x		
Physical therapy received				e				e
Resource use over 12 months								x

f = FOCUS, a = AFFINITY, e = EFFECTS, x = completed in all three trials

At early follow-up, during the index admission and in the first month to identify adverse events, monitor adherence is carried out by the local centres in all three trials. However, each trial’s national coordinating centres follow up the patients at 6 and 12 months with postal and telephone questionnaires to measure the primary and secondary outcomes. Data are also collected from general practitioners and by data linkage to mortality and hospital admission data in all three trials. The reasons why patients stopped taking the trial medication will be recorded.

### Primary and secondary outcomes

The primary outcome is functional status, measured with the modified Rankin scale (mRS) [26] at the 6-month follow-up. We are using the simple modified Rankin scale questionnaire [31–33] delivered by postal questionnaire, or via interview over the telephone or face to face to determine the mRS.

Secondary outcomes

- Survival till the end of the trial. This will be determined by following patients up for 12 months through their GPs and telephone and postal questionnaire and thereafter through linkage to routine mortality data
- Functional status (mRS) at 12-month follow-up
- Health status with the Stroke Impact Scale (SIS) (for each of nine domains on which the patient scores 0–100) [34–36]
- Arm, hand, leg and foot strength
- Hand function
- Mobility
- Communication and understanding
- Memory and thinking
- Mood and emotions
- Daily activities
- Participation in work, leisure and social activities
- Overall rating of recovery
- Adverse events/outcomes
- Depression. Although the SIS includes a domain reflecting mood, in the trials we are collecting additional information on the diagnosis and treatment of depression during follow-up. Participants are asked if they have been diagnosed with depression since their last assessment, whether this has been treated and whether they have been started on an antidepressant medication. Mood is assessed during follow-up in FOCUS and EFFECTS with the Mental Health Inventory 5 [37–39]. In addition EFFECTS uses the Montgomery-Åsberg Depression Rating Scale (MADRS) and patients scoring high have a diagnosis of depression confirmed based on the DSM-IV criteria [40–42]. The PHQ-9 [43] is administered at baseline (covering the 4 weeks before stroke), 1, 3, 6 and 12 months in AFFINITY
- Recurrent stroke including ischaemic and haemorrhagic strokes
- Acute coronary syndromes
- Epileptic seizures
- Episodes of hyponatraemia (<125 mmol/l)
- Upper gastrointestinal bleeding
- Other major bleeds (lower GI, extracranial, intracranial but extracerebral)
- Poorly controlled diabetes including hyperglycaemia (>22 mmol/l) and hypoglycaemia
- Falls resulting in injury
- New fractures
- Attempted suicide/self harm
- Death
- Fatigue (Vitality subscale of SF36) [44, 45]
- Cognition—the SIS, which incorporates an assessment of memory and thinking, is used for all three trials. In AFFINITY cognition during follow-up is assessed with the Modified Telephone Interview for Cognitive Status (TICSm) [46]. EFFECTS assess cognition with the Montreal Cognitive Assessment (MoCA) [47]
- Health-related quality of life measured with the five-level Euroqol 5D (EQ5D-5 L) to generate utilities [48]

Each trial is collecting data about resource use over the first 12 months to enable us to carry out health economic analyses.

### Provisional analysis plan

A detailed analysis plan for each trial, and for an individual patient data meta-analysis, will be developed and reported by the chief investigators and an independent statistician prior to the database being locked at the end of follow-up for final analysis.

The primary analyses will retain patients in their original assigned treatment groups. We will use ordinal regression to compare functional status (mRS scores) at the 6-month follow-up, adjusted for those factors included in our minimisation algorithm [49].

In secondary analyses we will compare the two treatment groups with respect to the following outcomes at 6 and 12 months:

- Survival will be analysed with the Cox proportional hazards model adjusting for the factors included in the minimisation algorithm

There is evidence that functional outcome at 6 months post stroke is strongly associated with long-term survival [50]. Therefore, if fluoxetine treatment is associated with improvements in functional status at 6 months it would be important to establish whether this translates into longer survival.

### Subgroup analyses

The functional status (mRS) at 6 months will be compared with ordinal regression in the following subgroups:

- Age (≤70, > 70 years)
- Baseline probability of a good outcome on mRS calculated with the six simple variable model [27]—to see whether effects remain constant across the range of stroke severities (<0.15 vs. 0.15-1 probability of being alive and independent at 6 months)
- Ischaemic vs. haemorrhagic stroke
- Patients who were unable to consent for themselves since this subgroup will allow us to answer the question whether routine use of fluoxetine is likely to benefit patients in whom a formal assessment of mood is impossible because of communication and cognitive problems.

In addition we are particularly interested to know whether the effect of treatment on neurological function is modified by specific neurological deficits present at baseline. Because patients may have a combination of neurological deficits, individual patients may appear in more than one subgroup

- Subgroup 1: Patients with a motor deficit affecting the face/arm or leg
  - Relevant outcomes: SIS-Strength, mobility, hand/arm function

- Subgroup 2: Patients with aphasia
  - Relevant outcomes: SIS-communication

The functional status (mRS) at 12 months will be compared with that at 6 months to establish whether any benefits observed at 6 months are maintained. If no difference is observed in the functional status between the treatment groups at 6 months, then secondary analyses will aim to establish whether there are differences with respect to the secondary outcomes, and if so whether these are maintained at 12 months.

We will also perform analyses of potential mediating factors, e.g. the role of depression. We will seek to answer the question whether any benefits are mediated by improvement in mood (based on MHI5, MADRS or PHQ9) and also whether any apparent loss of benefits in mRS or SIS between 6 months to 12 months is because of a deterioration in mood.

We envisage that levels of missing data in the primary outcome will be exceedingly low from previous experience of acquiring the mRS by postal and telephone questionnaire [51–55] and the primary analysis will be a complete case analysis. If we see higher levels of missing data than expected, we will use a suitable analysis, based on the likely missing data mechanism. We will consider whether to extend missing data methods to secondary outcomes at a blinded review of the Statistical Analysis Plan immediately before the database lock.

### Economic analyses

Each trial will develop a detailed health economic analysis plan before completion of data collection to determine the cost-effectiveness of fluoxetine in the trial setting.

### Sample size/power calculations

FOCUS, AFFINITY and EFFECTS are planning to enrol at least 3000, 1600 and 1500 patients respectively. Table 3 shows the effect sizes [expressed as a common odds ratio (COR)]. These numbers would provide 90 % power with an alpha of 0.05. These estimates are based on the distribution of outcomes in the seven categories of the mRS (0–6) (observed in both treatment groups combined amongst the first 451 patients enrolled and followed up at 6 months in the FOCUS trial).

**Table 3:** Sample size calculations derived from an ordinal regression and based on 90 % power and alpha 0.05

Trial	Sample size	Common odds ratio	% mRS 0–2 fluoxetine	% mRS 0–2 placebo	Absolute % improvement in mRS 0-2
EFFECTS	1500	1.35	49.5	42.1	7.4
AFFINITY	1600	1.34	49.4	42.2	7.2
FOCUS	3000	1.23	48.4	43.2	5.2
Pooled	4500	1.19	48.0	43.6	4.4
Pooled	6000	1.16	47.7	44.0	3.7

If FOCUS, AFFINITY and EFFECTS combined enrol 6000, we would have 90 % power (alpha 5 %) to detect a COR of 1.16, equivalent to a 3.7 % absolute difference in percentage with mRS 0–2 (44.0 % to 47.7 %).

The trial steering committees (TSC) will regularly review the target sample size and adjust this based on accruing blinded data on

- the enrolment into specific pre-specified subgroups
- completeness of follow-up
- distribution of mRS categories in the population of enrolled subjects (i.e. both treatment groups combined), overall and in specific patient categories (e.g. those with motor deficits, aphasia, etc.)

For example, if the distribution of mRS is different from that anticipated, then the sample size might need to be increased to maintain the power of the trial to detect the specified effect size. This approach has the advantage that such sample size adjustments can be made without reference to the accumulating blinded data and avoid the need for conditional power calculations, which can be unreliable.

### Study funding

The three trials are funded by grants from charitable organisations and government funding bodies (see acknowledgements for details). They do not receive any funding from the pharmaceutical industry.

### Ethics approvals

Each trial has received approval for its protocol and trial materials from the relevant local ethics committees and regulatory authorities in their respective countries [FOCUS: Scotland A Research Ethics Committee (for UK) (21/12/2011); AFFINITY: Western Australia, Royal Perth Hospital Human Research Ethics Committee (HREC) (24/02/2012), New South Wales, Victoria & Queensland, Western Sydney Local Health District HREC (30/04/2013), South Adelaide Clinical HREC (01/09/2014), New Zealand, Central Health and Disability Ethics Committee (17/04/2014)]. EFFECTS: Stockholm Ethics Committee (30/09/2013). No centres can start recruitment until they have received relevant ethics and regulatory approvals. Informed consent is obtained before the patient is enrolled except where it has been waived. Consent procedures had to comply with national requirements, so that in FOCUS and AFFINITY approval was obtained for consent by either the patient or proxy, AFFINITY also has approval for waiver of consent, whilst in EFFECTS patients have to be capable of consenting for themselves. The intensity and methods of monitoring were agreed between trial investigators and the relevant organisation in each country.

### Organisation

Each trial has established its own Trial Steering Committee (TSC) to oversee the conduct and progress of the trial. Each trial has its own independent Data Monitoring Committee (DMC) to oversee the safety of participants in the trial. During recruitment, interim analyses of the baseline and follow-up data will be supplied, in strict confidence, to the chairmen of the data-monitoring committees, along with any other analyses that the committees may request. In the light of these analyses, the data-monitoring committee will advise the chairmen of the Trial steering committees whether, in their view, the randomised comparisons have provided (1) 'proof beyond reasonable doubt' that for all, or some, the treatment is clearly indicated or clearly contra-indicated and (2) evidence that might reasonably be expected to materially influence future patient management. Following a report from the DMC, the steering committees will decide whether to modify entry to the study (or seek extra data). Unless this happens, however, the TSC, the collaborators and central administrative staff will remain ignorant of the interim results.

The terms of reference of the DMCs specify that any of the chairmen should confer with the chairmen of the DMCs of the other trials if they have concerns about the accruing data. The chairmen may elect to share blinded or unblinded reports and to request combined analyses of accruing data in the three trials.

The FOCUS trial is coordinated from the Edinburgh Clinical Trials Unit (ECTU) (Neurosciences Division) and sponsored by ACCORD (a joint organisation between University of Edinburgh and NHS Lothian). The AFFINITY trial is coordinated from the Stroke Research Unit in Perth (School of Medicine and Pharmacology, University of Western Australia, and Sir Charles Gairdner Hospital, Health Department of Western Australia) and the Neurological & Mental Health Division, George Institute for Global Health, in Sydney, Australia. The EFFECTS trial is coordinated from the Karolinska Institutet in Stockholm but its randomisation and data storage are hosted by the ECTU in Edinburgh.

### Discussion

The investigators of these three trials agreed to a collaborative approach including adhering to a common core protocol and combining their results to allow a prospective individual patient data meta-analysis. We judged that a larger number of participants would be required to answer our secondary research questions than could be recruited in any trial within a reasonable time period if based in one country. Also, we were keen to have sufficient power to ensure we detected even a modest overall effect, since this would still have important implications for patients, their families, and health and social services. The degree of collaboration is illustrated by the fact that the co-chief investigators of each trial participate in each of the three trial steering committees and have, where allowed, been named as co-applicants on each of the funding applications. This means that each of us has an interest in each of the three trials succeeding.

An alternative strategy that we considered was to establish a single international trial. However, we decided that our chosen strategy has several advantages.

We are able to vary trial procedures to fit local conditions. For instance, in the UK, the FOCUS trial is supported by a well-organised network of principal investigators and research staff in local centres funded by the National Institute of Health Research. In Australia and Sweden the individual trialists have to identify the local support networks. Regulations vary between countries with respect to whether proxy consent is acceptable and how patient identifiable data can be shared between centres and coordinating centres, which influences the feasibility of centralised follow-up and the intensity of monitoring required by regulators.

Ideally, the three trials would have collected identical data items at the same time points. Since the vast majority of data items are common to all three trials our approach did allow for some variation. For instance the PHQ9 was favoured in Australasia whilst the MHI5 was favoured in the UK and the MADRS in Sweden. These small variations should not preclude a valid combined analysis. Most importantly the eligibility criteria, method of randomisation, interventions and principal outcome measures were almost identical.

Our model allows us to confirm the results of one trial in another two providing valuable evidence of external validity. It will also allow us to explore any heterogeneity in trial results that may reflect the settings in which each trial was carried out. For instance, the intensity of background physical therapies, adherence to the trial drug, duration of hospitalisation and costs of delivering hospital and social services vary between countries and may alter the effectiveness or cost effectiveness of fluoxetine in treating stroke patients.

Our model negates the need to gain approvals for transporting supplies of active drug and placebo across international jurisdictions. It allowed the investigators in each country to apply for, and secure, funding for their own trial, which has provided more resources than were likely to be available from any one funding agency. Governmental and charitable funding agencies often put limits on how much of their grant funding can be spent abroad. Our approach also encouraged local ownership of the trial, which will hopefully facilitate faster recruitment and more complete follow-up.

Our DMCs are also collaborating to maximise patient safety. If safety concerns are raised as data accrue within one trial, the DMC charters encourage the chairs to share data for confirmation of a problem or for reassurance.

We urgently need new treatments to reduce the burden of disability after stroke. These three multicentre, investigator-led, charity- and government-funded randomised trials will determine whether the routine administration of fluoxetine 20 mg daily (for 6 months) in stroke survivors between 2 and 15 days after stroke improves recovery at 6 months, whether any benefits persist after fluoxetine has been discontinued and whether there are benefits in specific subgroups. If fluoxetine is effective, the results of the trial could be rapidly implemented throughout the world at very little cost to health services.

## Trials status

All three trials are actively recruiting. FOCUS recruited its first patient on 10 September 2012, AFFINITY on 11 January 2013 and EFFECTS on 20 October 2014. Our target is to complete recruitment in all three trials by 2018.

## Additional file

Additional file 1:
**Appendix.** Membership of collaborative groups. (DOCX 22 kb)

## Abbreviations

AFFINITY: Assessment oF FluoxetINe In sTroke recoverY
CI: Confidence intervals
COR: Common odds ratio
CTIMP: Clinical Trial of an Investigational Medical Product
DMC: Data monitoring committee
DSM IV: Diagnostic and Statistical Manual of Mental Disorders, version IV
ECTU: Edinburgh Clinical Trials Unit
EFFECTS: Efficacy oF Fluoxetine – a randomisEd Controlled Trial in Stroke
eGFR: estimated glomerular filtration rate
EQ5D-5 L: EuroQol 5 dimension questionnaire - five level
FLAME: Fluoxetine for motor recovery after acute ischaemic stroke
FOCUS: Fluoxetine Or Control Under Supervision
MADRS: Montgomery-Asberg Depression Rating Scale
MAOI: Monoamine oxidase inhibitor
MHI5: Mental Health Inventory 5 questions
MoCA: Montreal Cognitive Assessment
mRS: modified Rankin scale
NIHSS: National Institute of Health Stroke Scale
PHQ: Patient health questionnaire
RR: Relative risk
SF12 & 36: Short form 12 & 36
SMD: Standardised mean difference
smRSq: Simplified modified Rankin scale questionnaire
SIS: Stroke Impact Scale
SSRI: Selective serotonin reuptake inhibitor
TICSm: Modified Telephone Interview for Cognitive Status
TSC: Trial steering committee
