# Supplementary material for: The FOCUS, AFFINITY and EFFECTS trials studying the effect(s) of fluoxetine in patients with a recent stroke: a study protocol for three multicentre randomised controlled trials
Source: Trials. 2015 Aug 20;16:369. doi: 10.1186/s13063-015-0864-1 (PMC4545865; doi:10.1186/s13063-015-0864-1)
Supplement: Additional file 1: — Appendix. Membership of collaborative groups. (DOCX 22 kb) [file 13063_2015_864_MOESM1_ESM.docx]

**Appendix. Membership of collaborative groups**

**FOCUS**

| Name | Role | | Affiliation |
| --- | --- | --- | --- |
| **Trial Management Group** | | | |
| Martin Dennis | Co - CI | University of Edinburgh | |
| Ann Deary | Team member | University of Edinburgh | |
| Jonathan Drever | Data manager | University of Edinburgh | |
| Ruth Fraser | Team member | University of Edinburgh | |
| Catriona Graham | statistician | University of Edinburgh | |
| Karen Innes | Trial manager | University of Edinburgh | |
| Connor McGill | IT developer | University of Edinburgh | |
| Aileen McGrath | Team member | University of Edinburgh | |
| Gillian Mead | Co-CI | University of Edinburgh | |
| Alan Walker | IT lead | University of Edinburgh | |
| Pauli Walker | Team member | University of Edinburgh | |
| Carol Williams | Recruitment coordinator | University of Edinburgh | |
| **Pre HTA Steering Committee** | | | |
| Martin Dennis | Co-CI | University of Edinburgh | |
| John Forbes | Heath Economist | University of Limerick | |
| Maree Hackett | AFFINITY | The George Institute | |
| Graeme Hankey | AFFINITY | The University of Western Australia | |
| Karen Innes | Trial Manager | University of Edinburgh | |
| Steph Lewis | Statistician | University of Edinburgh | |
| Gillian Mead | Co-CI | University of Edinburgh | |
| Veronica Murray (dec) | EFFECTS | Karolinska Institutet | |
| Peter Sandercock | Chair | University of Edinburgh | |
| Judith Williamson | NIHR Patient rep |  | |
| **HTA Steering Committee** | | | |
| David Burgess | Independent NIHR lay Member |  | |
| Martin Dennis | Co CI | University of Edinburgh | |
| Jonathan Emberson | Independent Member | Oxford University | |
| Graham Ellis | Independent Member | NHS Lanarkshire | |
| Karen Innes | Trial manager | University of Edinburgh | |
| Gillian Mead | Co CI | University of Edinburgh | |
| David Stott | Independent Chair | Glasgow University | |
| Pippa Tyrrell | Independent Member | University of Manchester | |
| Judith Williamson | NIHR Patient rep |  | |
| Maree Hackett | AFFINITY | The George Institute | |
| Graeme Hankey | AFFINITY | The University of Western Australia | |
| Erik Lundström | EFFECTS | Karolinska Institutet | |
| Various | Sponsor Rep | ACCORD | |
| ***Independent Data Monitoring Committee*** | | | |
| Peter Langhorne | Chair | Glasgow university | |
| Fiona Reid | Statistician | St Georges, University of London | |
| Helen Rodgers | Stroke physician/trialist | Newcastle University | |
| ***Grant Holders (either Stroke Association or HTA)*** | | | |
| Craig Anderson | AFFINITY | The George Institute | |
| Martin Dennis | Co-CI | University of Edinburgh | |
| John Forbes | Health economics | University of Limerick | |
| Maree Hackett | AFFINITY | The George Institute | |
| Graeme Hankey | AFFINITY | The University of Western Australia | |
| Alan House | Psychiatry | University of Leeds | |
| Stephanie Lewis | Statistics | University of Edinburgh | |
| Malcolm Macleod | in vivo data and link to the Scottish Stroke Research Network | University of Edinburgh | |
| Gillian Mead | Co-CI | University of Edinburgh | |
| Daniel Morales | General practice | University of Dundee | |
| Peter Sandercock | Trialist | University of Edinburgh | |
| Frank Sullivan | General practice | University of Toronto | |

**AFFINITY**

| Name | Role | | Affiliation |
| --- | --- | --- | --- |
| **Trial Management Group** | | | |
| Sarah Barrett-Claxton | Trial office coordinator |  | |
| Anne Claxton | Office/nurse coordinator |  | |
| Maree Hackett | Co CI |  | |
| Graeme Hankey | Co CI |  | |
| Julia O’Dea | Office/nurse coordinator |  | |
| Michelle Tang | Trial office coordinator |  | |
| Clare Williams | Trial office coordinator |  | |
| **Steering Committee** | | | |
| Professor Osvaldo Almeida | Psychiatrist | University of Western Australia | |
| Craig S. Anderson | Geriatrician | George Institute for Global Health | |
| Christopher Etherton-Beer | Geriatrician | University of Western Australia | |
| Laurent Billot | Statistician | George Institute for Global Health | |
| Martin Dennis | FOCUS | University of Edinburgh | |
| Leon Flicker | Gerontologist | Royal Perth Hospital | |
| Andrew Ford | Psychiatrist | University of Western Australia | |
| Maree Hackett | Co-CI | The George Institute | |
| Graeme Hankey | Co-CI | The University of Western Australia | |
| Stephen Jan | Health economist | George Institute for Global Health | |
| Erik Lundström | EFFECTS | Karolinska Institutet | |
| Gillian Mead | FOCUS | University of Edinburgh | |
| Veronica Murray (dec) | EFFECTS | Karolinska Institutet | |
| ***Independent Data Monitoring Committee*** | | | |
| Gregory Carter | Psychiatrist | University of Newcastle | |
| Geoffrey Donnan | Neurologist | Florey Institute of Neurosciences & Mental Health | |
| Robert Hebert | Independent Chair | Neuroscience Research Australia | |
| Qilong Yi | Unblinded statistician | University of Toronto | |
| Qiang Li | Unblinded statistician | The George Institute | |
| ***Grant Holders*** | | | |
| Professor Osvaldo Almeida | Psychiatrist | University of Western Australia | |
| Christopher Etherton-Beer | Geriatrician | University of Western Australia | |
| Laurent Billot | Statistician | George Institute for Global Health | |
| Martin Dennis | FOCUS | University of Edinburgh | |
| Leon Flicker | Gerontologist | Royal Perth Hospital | |
| Andrew Ford | Psychiatrist | University of Western Australia | |
| Maree Hackett | Co-CI | The George Institute | |
| Graeme Hankey | Co-CI | The University of Western Australia | |
| Stephen Jan | Health economist | George Institute for Global Health | |
| Erik Lundstrom | EFFECTS | Karolinska Institutet | |
| Gillian Mead | FOCUS | University of Edinburgh | |

**EFFECTS**

| Name | Role | | Affiliation |
| --- | --- | --- | --- |
| **Trial Management Group** | | | |
| Eva Isaksson | Trial manager | Karolinska Institutet | |
| Erik Lundström | Chief Investigator | Karolinska Institutet | |
| Björn Mårtensson | Psychiatrist | Karolinska Institutet | |
| Per Näsman | Statistician | KTH Royal Institute of Technology | |
| **Steering Committee** | | | |
| Martin Dennis | FOCUS | University of Edinburgh | |
| Maree Hackett | AFFINITY | The George Institute | |
| Graeme Hankey | AFFINITY | The University of Western Australia | |
| Erik Lundström | EFFECTS | Karolinska Institutet | |
| Björn Mårtensson | Psychiatrist | Karolinska Institutet | |
| Gillian Mead | FOCUS | University of Edinburgh | |
| Veronica Murray (dec) | EFFECTS | Karolinska Institutet | |
| Per Näsman | Statistician | KTH Royal Institute of Technology | |
| Katharina Stibrant Sunnerhagen | Independent Chair | University of Gothenburg | |
| Björn Berman |  | Previously Medical Products Agency | |
| Bo Norrving | Professor | Lund University | |
| Per Wester | Professor | Umeå University | |
| Håkan Wallén | Professor | Karolinska Institutet | |
| Jörgen Borg | Professor | Karolinska Institutet | |
| Christina Sjöstrand | Ass. Professor | Karolinska Institutet | |
| **Independent Data Monitoring Committee** | | | |
| Kjell Asplund | Independent Chair | Umeå University | |
| Anders Ljungström | Independent Statistician |  | |
| Andreas Terént | Professor | Uppsala University | |
| **Grant Holders** | | | |
| Erik Lundström | EFFECTS | Karolinska Institutet | |
| Veronica Murray (dec) | EFFECTS | Karolinska Institutet | |
